# Neural Basis of Response Bias on the Stop Signal Task in Misophonia

**DOI:** 10.3389/fpsyt.2019.00765

**Published:** 2019-10-23

**Authors:** Nadine Eijsker, Arjan Schröder, Dirk J. A. Smit, Guido van Wingen, Damiaan Denys

**Affiliations:** ^1^Department of Psychiatry, Amsterdam Neuroscience, Amsterdam UMC, University of Amsterdam, Amsterdam, Netherlands; ^2^Amsterdam Brain and Cognition, University of Amsterdam, Amsterdam, Netherlands

**Keywords:** misophonia, functional magnetic resonance imaging, stop signal task, response bias, perfectionism, compulsivity

## Abstract

**Objective:** Misophonia is a newly described condition in which specific ordinary sounds provoke disproportionately strong negative affect. Since evidence for psychobiological dysfunction underlying misophonia is scarce, we tested whether misophonia patients, like many patients with impulse control or obsessive-compulsive spectrum disorders, show impaired ability to inhibit an ongoing motor response.

**Methods:** We collected functional magnetic resonance imaging data during a stop signal task in 22 misophonia patients and 21 matched healthy controls.

**Results:** Compared to controls, patients tended to show longer stop signal delays, which is the time between stimuli signaling response initiation and inhibition. Additionally, patients tended to activate left dorsolateral prefrontal cortex more during responding rather than successful inhibition, as was seen in controls. Furthermore, patients lacked inhibition success-related activity in posterior cingulate cortices and activated the superior medial frontal gyri less during inhibition success compared to failure, a feature correlated with stop signal delays over the sample.

**Conclusions:** Misophonia patients did not show impaired response inhibition. However, they tended to show a response bias on the stop signal task, favoring accuracy over speed. This implies perfectionism and compulsive, rather than impulsive, behavior. Moreover, brain activations were in line with patients, compared to controls, engaging more cognitive control for slowing responses, while employing more attentional resources for successful inhibition.

## Introduction

Misophonia is a newly described psychiatric condition in which specific ordinary sounds, such as breathing or lip-smacking, provoke disproportionately strong feelings of irritability, anger, and/or disgust (1–3). These symptoms often cause patients to experience anticipatory negative affect and the feeling of loss of self-control, making them react aggressively to their environment or avoid situations associated with the sound(s), resulting in problems in social and/or professional domains (1, 2). Across patients, there is a striking consistency in symptoms, trigger sounds, and coping mechanisms, which suggests that misophonia might be a discrete mental disorder (2, 4). However, misophonia has not yet been recognized by contemporary classification systems. As such, there is little awareness about this condition by the general public, health-care providers, and patients themselves.

Hence, evidence for psychobiological dysfunction is limited due to lack of research. Symptom provocation studies have found hyper-activated insular, anterior cingulate, and auditory cortex, as well as hyper-connectivity between insula and ventromedial prefrontal cortex, posteromedial cortex, hippocampus, and amygdala (5, 6). Furthermore, patients exhibited diminished N1 event-related potentials in response to auditory oddball tones (7). These findings thus support recognition of misophonia as a discrete disorder. Misophonia symptoms exhibit features of impulsivity and compulsivity, as do impulse control and obsessive-compulsive spectrum disorders. However, endophenotypes—measurable traits that exist between clinical phenotype and underlying genetic basis—are less heterogeneous across disorders than clinical features and therefore considered a superior aid for diagnostic classification and gaining insight into the neurobiology of psychopathology (8, 9). An endophenotype shared by many disorders featuring impulsive or compulsive behaviors is impaired response inhibition (8, 10–12). Response inhibition is the ability to inhibit an ongoing motor response in the face of changing demands. It is considered an executive function important for behavioral flexibility and therefore adaptive behavior in a changing environment. Response inhibition failure is referred to as impulsivity. (Dysfunctional) impulsivity, behaviorally reflected in speedy and non-reflective decisions and resulting in negative consequences, shows a positive correlation with anger levels (13), which have previously been found elevated in misophonia (6). Moreover, males with high trait aggression have been found to show impaired response inhibition in a socio-emotional context, behavior that was accompanied by attenuated activation of inhibition-related brain areas (14). Both impaired response inhibition and (trait) anger/aggression may thus contribute to impulsive aggression, posing the question to what extent impulsivity underlies misophonia symptoms.

A frequently adopted paradigm to study response inhibition is the stop signal task (SST), which requires balancing speed (rapid response to a go-signal initiating action) and accuracy (successful inhibition of an ongoing response following a stop signal). The task is theoretically grounded in the horse-race model (15), which posits that response inhibition depends on the relative “finishing” times of independent and competing “go” and “stop” processes. The SST manipulates the delay in appearance of the go and stop-stimuli (stop signal delay or SSD), making it easier (short delay) or harder (long delay) to inhibition the response. This manipulation enables approximation of an individual's stopping latency (stop signal reaction time or SSRT), which is considered an indicator of response inhibition ability.

To improve our understanding of underlying neurobiological mechanisms and further substantiate recognition of misophonia as a distinct disorder, we tested whether misophonia patients show impaired response inhibition. We measured blood-oxygen-level dependent (BOLD) responses during SST performance using event-related functional magnetic resonance imaging (fMRI) in 22 misophonia patients and 21 age, sex, and education level-matched healthy controls. We tested if patients and controls show different (1) SSRTs, SSDs, or reaction times (RTs), or (2) brain responses during SST performance, and (3) how such potential differences are associated with each other.

## Materials and Methods

### Participants

Twenty-five misophonia patients were recruited from the Academic Medical Center (AMC) outpatient clinic. Twenty-five controls, matched on age, sex, and education level, were recruited *via* advertisements at the AMC/University of Amsterdam. Patients were diagnosed on the basis of the criteria postulated by Schröder et al. (2) by three AMC psychiatrists experienced in diagnosing misophonia. All patients experienced anger and a subset additionally experienced disgust in reaction to eating sounds and at least three of the following sounds: heavy breathing/sniffling, keyboard typing, chewing, and slurping. Participants were interviewed by another psychiatrist (AS) to assess (additional) misophonia symptoms, psychiatric diagnoses, current and previous health issues, medication use, alcohol or substance use, and handedness. Exclusion criteria included presence of major depression, anxiety disorder, bipolar disorder, psychotic disorder, autism spectrum disorder, substance related disorder, hearing loss, epilepsy, structural central nervous system disorder, stroke within the last year, and MRI contraindications.

One patient reported misophonia symptoms with only two out of four sounds but was also included because of severe misophonia. Two patients had co-morbid attention deficit (hyperactivity) disorder, of whom one used methylphenidate (30 mg daily), and one had a borderline personality disorder. In total, data from 1 patient and 2 controls were missing due to technical problems and 2 patients and 2 controls were excluded because of extremely poor task performance (see behavioral analysis section for exclusion criteria). Three patients and three controls were left-handed.

The study was approved by the Medical Ethics Committee of the AMC, Amsterdam, and all participants were explained the nature of the experimental procedures and subsequently provided written informed consent prior to inclusion in the study. The data reported here were obtained in a larger study in which we also collected resting-state fMRI, diffusion weighted imaging, and functional MRI during viewing of aversive movies. The results from the other experiments will be reported elsewhere.

### Questionnaires

All participants filled out Dutch versions of the following questionnaires: The Symptom Checklist [SCL-90(16)], which assesses general mental and physical dysfunction, the Hamilton Anxiety Rating Scale [HAM-A(17)] and Hamilton Depression Rating Scale [HAM-D(18)], which, respectively, assess anxiety and depressive symptoms, and the Buss-Perry Aggression Questionnaire [BPAQ(19, 20)], which assesses aggressive personality style by means of four categories: physical aggression, verbal aggression, anger, and hostility. The total score of the BPAQ is considered a general index of trait aggression. Misophonia severity was scored using the Amsterdam Misophonia Scale (A-MISO-S) (2).

### Stop Signal Task

**Figure 1:**
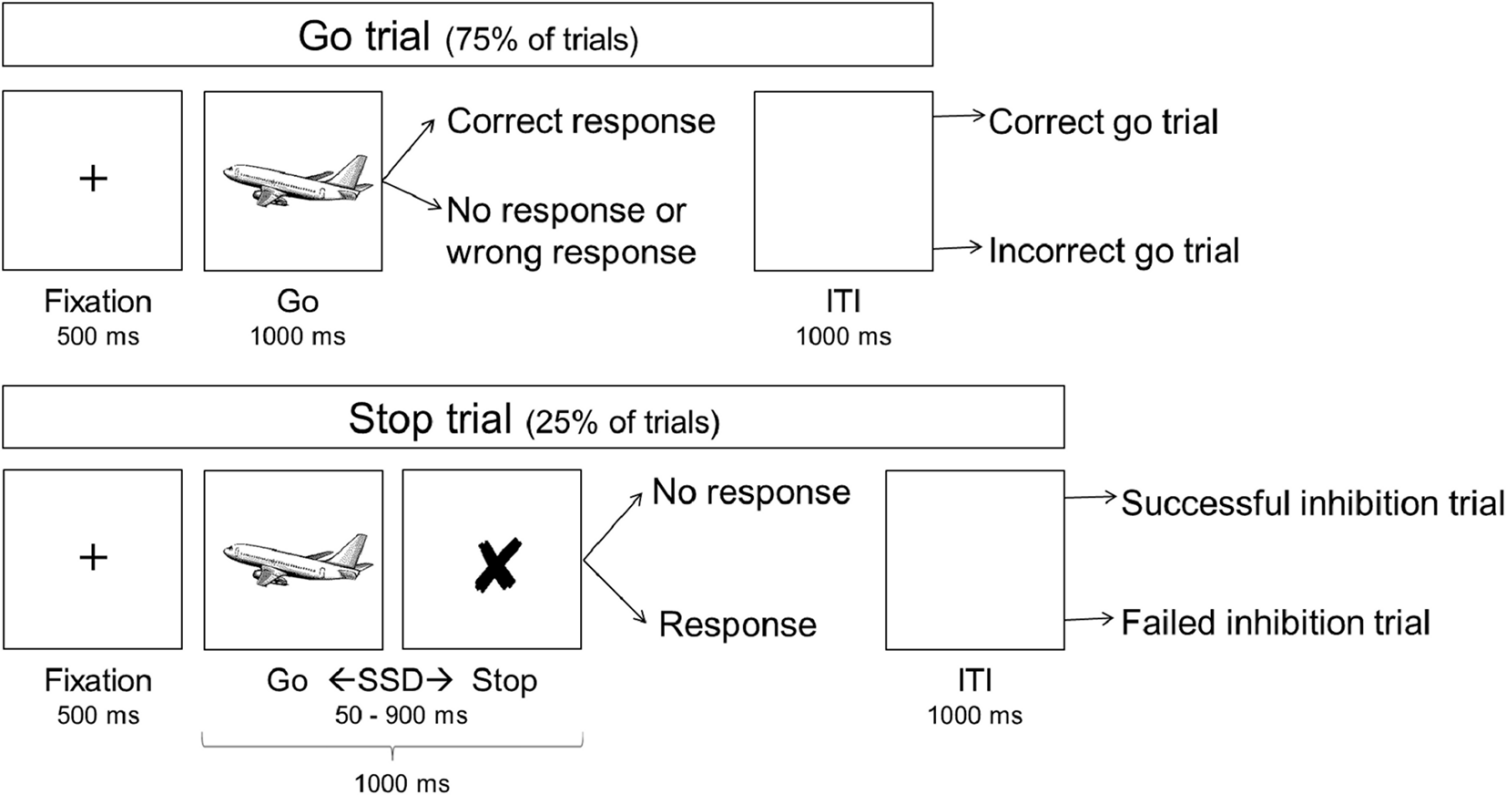
Schematic overview of the stop signal task. ITI, inter-trial interval; SSD, stop signal delay.

**Figure 1** shows a schematic overview of the SST, which consisted of 279 trials, each lasting 2,500 milliseconds (ms), including a 1,000 ms inter-trial-interval. All stimuli were black schematics centered on a white background. Each trial started with a fixation cross (500 ms), followed by an image of an airplane (go-signal; 1,000 ms on go trials) with its nose pointing either left or right, to which participants responded as quickly as possible by pressing the corresponding left of right button on a keypad using their dominant hand. On stop trials (25%), the go-signal was replaced with a cross (stop signal) after a variable delay, i.e. the stop signal delay (SSD). This signaled participants to inhibit the initiated response. The SSD started at 250 ms and a staircase procedure increased it by 50 ms after a successful stop trial, making inhibition on the subsequent stop trial more difficult, and decreased it by 50 ms after a failed stop trial, making inhibition on the subsequent stop trial easier. The staircase procedure is thought to result in an SSD that represents the delay required for a subject to successfully inhibit a response in approximately half of stop trials (21). All participants were explicitly told that speed on go trials and accuracy on stop trials were equally important.

### Behavioral, Demographic, and Clinical Data Analysis

Behavioral data were first processed in Matlab version R2014b (22), after which statistical analyses were performed in Statistical Package for the Social Sciences (SPSS) version 24 (23). Firstly, we selected 1) correct go trials, 2) incorrect go trials (lack of response or incorrect response), 3) successful stop trials, and 4) failed stop trials (**Figure 1**). Trials with negative RTs and trials in which a response to the go-signal preceded the stop signal were excluded.

SSRT was calculated using the quantile method (24), which has been shown less susceptible to violations of assumptions underlying the horse-race model than other methods. (24, 25) Per individual, the quantile reaction time (QRT) is calculated by sorting RTs on correct go trials ascendingly and calculating the RT corresponding to the quantile of the proportion of stop trials that failed. The QRT is the RT for which approximately half of the go trials were faster and the other half were slower. Subsequently, SSRT was obtained by subtracting SSD from the QRT. Behavioral measures of interest were SSRT—the main indication of response inhibition ability—and mean RTs on correct go and failed stop trials. In addition, SSD was looked at since it conveys information about inhibitory performance independently of RTs. For performance-based exclusion, we restricted the proportions of inhibition success (no less than 25% of stop trials) and failure (no more than 75% of stop trials) and correct go trials [no less than 60% of go trials (26)], resulting in the exclusion of two patients and two controls from analysis.

Next, data points that deviated more than three times the inter-quartile range (distance between the first and the third quartile) from the first or third quartile—in SPSS marked as an “extreme outlier”—were removed from the test. Group differences in demographical and clinical measures were tested for using independent samples t-tests for normally distributed measures, Mann-Whitney U-tests for non-normally distributed measures, and Chi-square tests for categorical measures. Group differences in behavioral measures were tested for using Analysis of Covariance (ANCOVA) to allow controlling for ranked age, sex, and handedness. To maintain an alpha of .05, significance thresholds were corrected for multiple comparisons using the Tukey-Ciminera-Heyse (TCH) method with the modification suggested by Sankoh et al. (27), which takes the covariance of the tested measures into account.

### MRI Data Acquisition

Anatomical and functional images were acquired using a Philips Ingenia 3.0 T MRI system (Philips Medical Systems, Best, The Netherlands) with a SENSE 32 elements head coil. To minimize movement artifacts, participants’ heads were fixed using foam padding. A whole-brain anatomical T1-weighted image (3D MP-RAGE) was acquired (voxel size = 1 mm^3^, TR/TE = 7000/3.2 ms, matrix = 256 × 256, field of view = 256 × 240 mm, 180 sagittal slices). Whole-brain functional images were acquired using transverse T2*-weighted Echo-Planar Imaging (EPI; TR/TE = 2,000/27 ms, matrix = 80 × 80, in-plane resolution = 3 × 3 mm, slice thickness = 3 mm, slice gap = 0.3 mm, 37 ascending slices), which uses a gradient-echo pulse sequence for detecting BOLD contrast.

### MRI Data Preprocessing

Imaging data were preprocessed and analyzed using statistical parametric mapping (SPM) version 12 (28) implemented in Matlab version R2014b (22). Functional images were corrected for head motion by alignment to the first image and then to the mean of all images using a six-parameter rigid-body transformation. All head-movement parameters remained within acceptable limits (<3.0mm). Afterwards, slice timing correction was applied to account for differences in acquisition time between slices. Next, the functional images were co-registered to the anatomical image and subsequently normalized to Montreal Neurological Institute space, resampled to 2 × 2 × 2 mm^3^, and spatially smoothed with a Gaussian kernel of 8mm full-width at half maximum.

### MRI Data Analysis

For each participant, the SST was modelled as an event-related design using the general linear model. The trials were modelled by convolving the onsets of the go stimuli with the canonical hemodynamic response function for the following conditions: 1) successful stop trials, 2) failed stop trials, and 3) a subset of randomly selected correct go trials. To keep approximately equal numbers of trials in each category, the number of randomly selected correct go trials was the mean of the number of available successful and failed stop trials. Additional task regressors of no interest consisted of the remainder of correct go trials, the incorrect go trials, and trials with negative reaction times. Data were high-pass filtered with a cut-off at 128 seconds to remove slow signal drifts and an AR(1) autoregression model accounted for serial correlations.

Group differences were tested using a group X condition mixed model ANCOVA, in which the images for the correct go, successful stop, and failed stop conditions were entered as repeated measures for each group separately. Sex, ranked age, and handedness were entered as covariates of no interest. Voxel-wise statistical tests were family-wise error (FWE) rate corrected for multiple comparisons at the cluster-level across the whole brain (p_FWE_ < .05; cluster-defining threshold p < .001), or across the regions of interest (ROI) at the peak-level using a small volume correction (p_SVC_ < .05). To account for testing multiple ROIs, an additional correction was applied, using the abovementioned modified TCH method, resulting in a threshold of p_FWE_ = .022.

Using the WFU PickAtlas tool (29), we constructed three ROIs that combined brain regions that were related in function: (1) a “control” ROI including left anterior cingulate cortex (ACC) and left DLPFC (30), (2) an “affect” ROI including right insula and right inferior frontal gyrus (IFG) (31), and (3) a “motor” ROI including bilateral pre-supplementary motor area (pre-SMA) and bilateral basal ganglia, including the caudate nuclei, putamens, and the sub-thalamic nuclei (32). The control and affect ROIs and the pre-SMA in the motor ROI were defined as spheres with 10mm radius around coordinates previously reported by fMRI studies examining the speed-accuracy trade-off (30) and response inhibition (31, 32). For the basal ganglia, the automated anatomical labeling atlas (33) was used to define the caudate nuclei and putamens and an atlas of subcortical structures (34) was used to define the sub-thalamic nuclei (sphere with 5 mm radius).

### Brain-Behavior Correlations

For the association between brain and behavior, the first principal component—or eigenvariate—for each condition was extracted per subject from the clusters that showed an interaction effect (volume of interest defined as “cluster”, with a cluster-defining threshold of p = .001). These values represent the “typical” participant response over the voxels within the cluster, without assuming homogenous responses. The eigenvariates were then imported into SPSS and the relevant contrasts were (re-)created in such a way that the resulting values represented the degree of patient’s neural aberration, i.e. either a surplus or a shortage of activity compared to controls. These values were then correlated with behavioral measures that differed between groups. Fisher’s r-to-Z transformation was used to test if brain-behavior correlations differed between groups. Outlier rejection and multiple comparison correction for these data were identical to that for the behavioral, demographic, and clinical data, as described above.

## Results

### Demographic and Clinical Characteristics

Fifteen out of 22 patients experienced disgust in addition to anger in response to trigger sounds. Patients and controls did not differ in age, sex, education level, or handedness (**Table 1**). For patients, symptoms emerged on average at the age of twelve and the average symptom severity scored as 15 out of a maximum of 40 (A-MISO-S). Patients scored significantly higher on general psychopathy (SCL-90), anxiety (HAM-A), depression (HAM-D), anger (BPAQ total score and anger subscale), and hate (BPAQ hate subscale).

**Table 1:** Demographic and clinical characteristics.

	Controls (N = 21)		Patients (N = 22)		Statistical analysis	
					Test statistic	*p*-value
**Sex** (female; N, %)	17 (81%)		16 (73%)		χ*²* = 0.41	.52
**Age** (years; mean, SD)	32.4 (10.0)		33.2 (9.6)		*U* = 246.0	.72
**Educational level** (median, range)**^†^**	6 (2–7)		6 (2–7)		χ*²* = 0.89	.83
**Handedness** (right-handed; N, %)	18 (86%)		19 (86%)		^§^	1.0
**Age of onset** (years; mean, SD)			11.9 (3.2)			
				
**Measures^‡^**	***Mean***	***SD***	***Mean***	***SD***		
**A-MISO-S**			15.0	2.7		
**SCL-90**	104.6	14.5	147.9	40.9	*U* = 344.5	.001^¶^
**HAM-A**	2.3	3.8	13.0	8.8	*U* = 359.5	<.001^¶^
**HAM-D**	1.7	2.6	9.3	6.0	*U* = 374.5	<.001^¶^
**BPAQ**						
**Physical Aggression**	16.6	3.3	18.5	3.7	*t* = 1.66	.10
**Verbal Aggression**	11.9	3.1	12.0	2.8	*t* = 0.05	.96
**Anger**	13.9	3.7	20.4	5.5	*U* = 368.5	<.001^¶^
**Hate**	14.2	4.3	20.2	7.8	*U* = 323.5	.009^¶^
**Total score**	56.9	9.7	72.1	17.2	*t* = 3.52	.001^¶^

N, number; SD, standard deviation; A-MISO-S, Amsterdam Misophonia Scale; SCL-90, Symptom Checklist; HAM-A, Hamilton Anxiety Rating Scale; HAM-D, Hamilton Depression Rating Scale; BPAQ, Bush Perry Aggression Questionnaire. ^†^Educational level was categorized using the 2011 ISCED system (UNESCO Institute for Statistics, 2012), ranging from 0 (no finished education) to 8 (doctorate obtained).^‡^Missing data: A-MISO-S: 1 patient, SCL90: 1 patient, 2 controls; HAM-A/HAM-D: 2 controls, BPAQ: 1 patient. ^§^Fisher's exact test (2-sided). ^¶^Significant after TCH correction with Sankoh et al. (27) modification (p < .009).

### Behavior

Over groups, accuracy rate for the go trials was 98% (range 91–100%) and the percentage of failed stop trials was 52% (range 47–58%). Patients and controls did not show differences in SSRT or RTs (**Table 2**). Patients exhibited longer SSDs than controls, which did not remain significant after multiple comparison correction.

**Table 2:** Performance on the stop signal task.

	Controls		Patients		Statistical analysis	
	Mean	SD	Mean	SD	Test statistic^†^	p-value
**SSRT** (ms)	191	69	160	51	*F* = 2.74	.106
**SSD** (ms)	321	145	406	124	*F = 4*.81	.035^‡^
**Correct go RT** (ms)	550	125	604	114	*F* = 2.76	.068
**Failed stop RT** (ms)	500	115	553	117	*F = 2*.63	.113
**Go accuracy** (% correct go trials)	97	2	98	2		
**Inhibition accuracy** (% successful stop trials)	51	2	52	2		

SSRT, stop signal reaction time, indicative of stopping latency; SSD, stop signal delay, i.e. time between go and stop signals; RT, reaction time. ^†^(df between = 1, df within = 38). ^‡^Did not survive TCH correction with Sankoh et al. (27) modification (p < .018).

SSD was highly correlated with correct go RTs [r(41) = 0.973, p < .001] and inhibition accuracy [r(41) = 0.893, p < .001] over groups. These correlations did not differ between groups (Z = 0.43, p = .67 and Z = 0.56, p = .58, respectively).

### fMRI

No main effects of group were found.

#### Successful Inhibition Versus Correct Going

Across groups, successful inhibition compared to correct going activated the right middle occipital gyrus extending to inferior temporal gyrus and angular gyrus, left middle-to-inferior occipital gyrus extending to fusiform gyrus, bilateral insula and superior frontal gyri, with the right insula cluster extending to inferior frontal gyrus, and left middle-to-superior frontal gyrus (**Supplementary Table 1**). Conversely, correct going compared to successful inhibition was associated with activation in the left pre and post-central gyri, bilateral caudate nuclei and cunei, left insula and bilateral rolandic opercula extending to superior temporal gyri, right cerebellum, and right pre-central gyrus.

**Figure 2:**
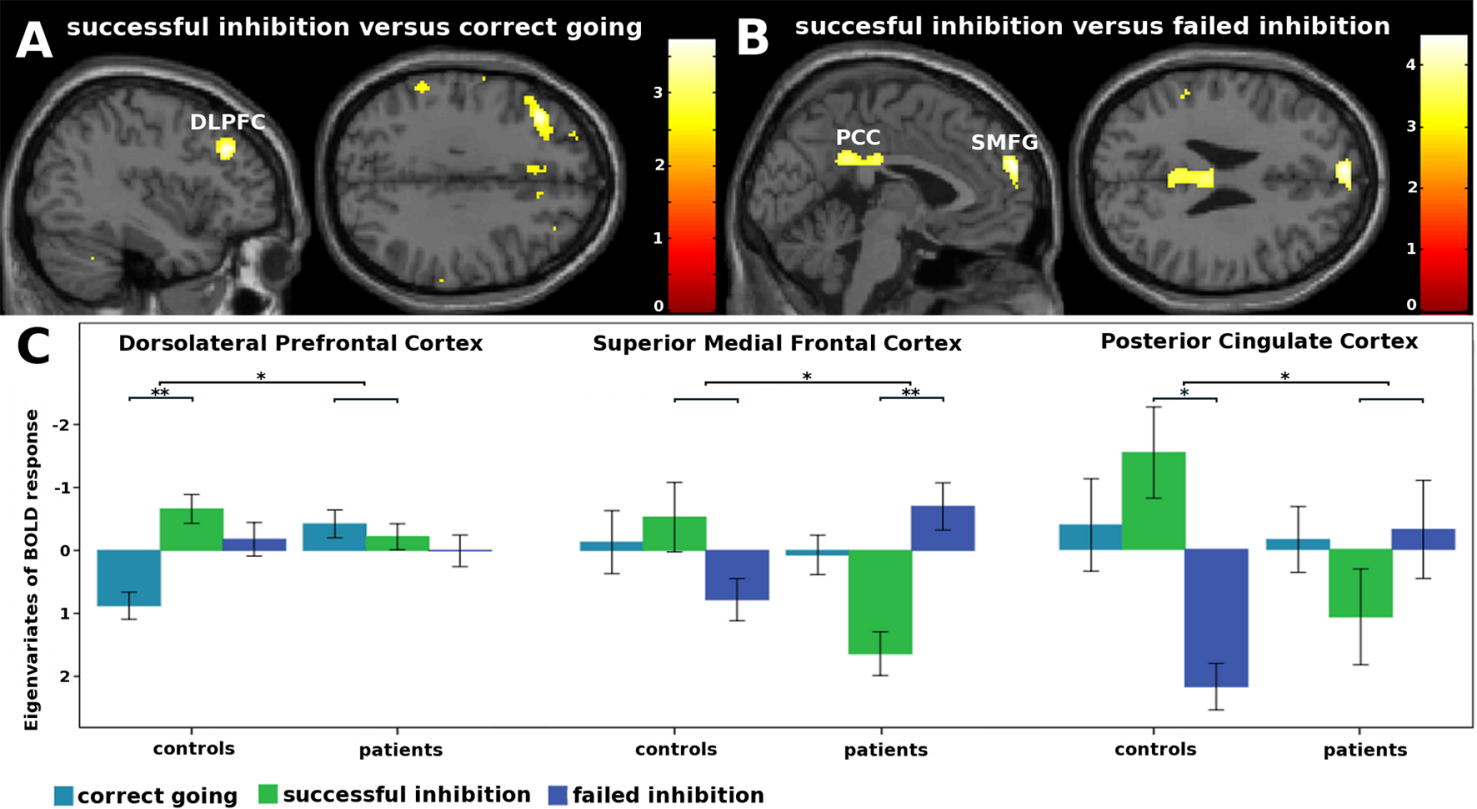
fMRI interaction effects. **(A)** Not only did patients lack the inhibition success-related activation of left dorsolateral prefrontal cortex that controls showed, they tended to activated this region more during correct going than during successful inhibition. **(B)** Patients activated the superior medial frontal gyri less during inhibition success compared to failure, whereas controls did not. Controls showed inhibition success-related activity in the posterior cingulate cortex, whereas patients did not. **(C)** Eigenvariates of BOLD responses (± 1 standard error of means) per condition for each of the clusters that showed an interaction effect. *p < .05. **p < .001.

For this contrast, a group by condition interaction was found in the left DLPFC (**Figure 2** and **Table 3**). Subsequent t-tests (**Supplementary Table 2**) showed that patients not only lacked the inhibition success-related activation of left DLPFC that controls showed T(1,41) = 5.19, Z = 4.92, p < .001, they tended to activate this region more during correct going than during successful inhibition, T(1,41) = 3.21, Z = 3.14, p = .068.

**Table 3:** fMRI interaction effects.

Region	Side	ROI	p-value	Voxels in cluster	Peak z-value	MNI coordinates		
**x**	**y**	**z**
**Successful inhibition versus correct go**								
*Inferior frontal gyrus (pars orbitalis)*	*R*	*Affect*	*.060*	–	*3.18*	*32*	*26*	–*6*
Dorsolateral prefrontal cortex	L	Control	.014^†^	–	3.66	–38	30	32
								
**Failed inhibition versus correct go**								
*Dorsolateral prefrontal cortex*	*L*	*Control*	*.063*	–	*3.17*	–*38*	*30*	*32*
								
**Successful versus failed inhibition**								
Superior medial frontal gyrus	L+R		.027^‡^	310	4.28	-2	58	26
Posterior cingulate cortex	L+R		.027^‡^	311	3.87	4	–26	28
*Insula*	*R*	*Affect*	*.088*	–	*3.04*	*38*	*10*	–*2*
*Inferior frontal gyrus(pars triangularis)*	*R*	*Affect*	*.093*	–	*3.00*	*38*	*28*	*2*

Clusters with a p-value between.05 and.10, without correction for number of ROIs, are printed in italic. ^†^Significant after small volume correction with peak-level FWE-corrected p < .05 with TCH correction with Sankoh et al. (27) modification for number of ROIs (p < 0.02). ^‡^Significant after whole-brain cluster-level FWE-corrected p < .05 with cluster-defining thresholding of p < 0.001.

#### Failed Inhibition Versus Correct Going

Across groups, failed inhibition compared to correct going activated the bilateral insula extending to inferior frontal gyrus (left) and pre-central gyrus (right), bilateral medial cingulate cortices extending to posterior medial frontal cortices, left supramarginal gyri extending to inferior and middle occipital gyri, right superior temporal gyrus, left pallidum extending to the pons (bilateral), and left calcarine gyrus (**Supplementary Table 1**). Conversely, correct going compared to failed inhibition activated the bilateral inferior frontal gyri, on the right side extending to the caudate nucleus, right cerebellum, left angular gyrus, right superior temporal gyrus extending to the dorsolateral prefrontal cortex (DLPFC), left parahippocampal gyrus/hippocampus, right superior temporal and occipital gyri, and left posterior cingulate cortex extending to the precuneus. No significant group by condition interactions were observed for this contrast.

#### Successful Versus Failed Inhibition

Across groups, successful compared to failed inhibition activated the left middle orbital gyrus extending to inferior frontal gyrus, right putamen, right angular gyrus extending to post-central gyrus, and right calcarine gyrus extending to middle and inferior occipital gyri (**Supplementary Table 1**). Trends were observed for left inferior parietal lobule extending to angular gyrus (p = .056) and right inferior-to-middle temporal gyrus (p = .052). Conversely, failed compared to successful inhibition activated the bilateral anterior-to-medial cingulate cortices, right pre-central gyrus extending to middle frontal gyrus, left post and pre-central gyri extending to superior temporal gyrus and the left temporal pole, bilateral calcarine gyri, and right rolandic operculum extending to the insula.

Importantly, group by condition interaction effects were found in the superior medial frontal gyri (SMFG) and posterior cingulate cortices (PCC; **Figure 2** and **Table 3**). Subsequent t-tests revealed that patients activated the SMFG less during inhibition success compared to failure, T(1,41) = 7.70, Z = 6.93, p < .001, whereas controls did not. Moreover, whereas controls showed inhibition success-related activity in the PCC, T(1,41) = 4.79, Z = 4.57, p = .048, patients did not.

### Brain-Behavior Correlations

Eigenvariates were extracted from the DLPFC, SMFG, and PCC clusters and correlated with SSD. Over groups, SSD correlated positively with SMFG response during failed compared to successful inhibition (r(41) = 0.38, p = .012), whereas no correlations were found for the DLPFC (r(41) = 0.29, p = .062) and PCC (r(40) = 0.29, p = .066) clusters. Correlations with SSD did not differ between groups for any of the clusters (**Supplementary Table 3**).

## Discussion

We tested response inhibition ability in misophonia patients and controls and found that patients tended to have longer SSDs than controls. Functional neuroimaging showed that patients activated the SMFG less during successful compared to failed inhibition, whereas controls did not. This pattern of activity was moderately positively correlated with SSD over the sample. Patients additionally lacked the inhibition success-related activation of the PCC that controls exhibited and they tended to activate the left DLPFC more during correct going than during successful inhibition, which was opposite to the pattern in controls.

### Response Bias

Patients might show a response bias on the SST, favoring accuracy over speed. That is, since inhibition success lengthens the SSD, the high correlation between SSD and RTs likely reflects the speed-accuracy trade-off. Patients showed both a tendency towards longer SSDs and (non-significantly) longer RTs, implying the slowing of responses in service of accuracy.

Healthy subjects are also known to pro-actively employ strategic slowing on the SST on a trial-by-trial basis in order to adaptively aid speed-accuracy balance. (15, 35–38) However, the current sample of patients seems to have shifted the balance beyond the point of speed-accuracy optimization and show a pattern opposite of that observed in more impulsive subjects, who favor speed over accuracy (13). Interestingly, behavior such as that currently found has previously been linked to less compliant/empathic personality traits in healthy subjects. (38) This appears in line with the high comorbidity of obsessive-compulsive personality traits in misophonia (2, 39, 40), which is likewise characterized by inflexible and strong coherence to one's "own rules". Indeed, the observed putative error-avoidant strategy could be explained by behavioral inflexibility or perfectionism/intolerance towards mistakes (41–43). Importantly, a similar response bias during proof-reading performance has been associated with low(er) efficiency and perceptions of consistently failing to meet one's own perfectionistic standards and expectations and associated negative emotions, including disappointment, frustration, and anxiety (44). Furthermore, self-oriented perfectionism shows a positive correlation with all—but particularly the depression, anxiety, and hostility—SCL-90 subscales (45), a questionnaire on which patients scored significantly higher than controls (6). Moreover, self-oriented perfectionism has been associated with trait anger and experience of negative effect, with the latter being mediated by anxiety sensitivity (46, 47). In addition to anger, disgust, and sadness (2, 6), some patients additionally report experiencing anxiety in response to triggers (1) and consistently show higher Hamilton Anxiety Scale scores than controls and the general population (2, 6). Their putative perfectionism thus seems to fit in well with the aggregate of symptoms and associated characteristics, including trait anger and anxiety sensitivity. Moreover, mental inflexibility has been linked to behavioral inflexibility, anxious, sad, and angry mood states, and avoidance, which is the most frequently reported coping strategy for misophonia (48, 49). Together with the current finding, this puts forward inflexibility as an interesting topic for future misophonia research.

### Aberrant Activation of the Left DLPFC, SMFG, and PCC

The left DLPFC has been implicated in strategy development and implementation by exerting cognitive control, including modification of speed-accuracy balance specifically (50–52). Patients' lack of inhibition success-related left DLPFC activation could thus signify two things. One is less employment of cognitive control for response inhibition, which is enabled by strategic waiting making inhibition less difficult. The other is less adjustment of behavioral policy—in this case shifting the speed-accuracy trade-off towards speed—after successful inhibition. Conversely, patients' tendency to activate left DLPFC more during correct going might signify exaggerated strategic slowing of responses.

Additionally, contrary to controls, patients seemed to generally activate the SMFG and PCC less during successful compared to failed inhibition. These areas are key nodes of the default-mode network, which deactivates during most cognitively demanding tasks—thought to reflect external focus of attention and stimulus-oriented processing, which is linearly scaled to task difficulty (53)—and activates during rest—thought to reflect introspection and stimulus-independent processing (54, 55). Specifically, the SMFG specifically deactivates more with increasing working memory load, irrespective of attentional demand, whereas the PCC deactivates more with increases in both (56). Hence, current misophonia patients may either have required greater engagement of working memory or focusing more attention to the task at hand to achieve (such frequent) successful response inhibition.

However, although the SMFG and PCC typically respond highly similarly during cognitive and emotional processing (55, 57), they can be distinguished by, for example, functional connectivity correlating positively and negatively, respectively, with depressive and anxiety symptoms. (58, 59) Since the SMFG has specifically been implicated in (social) reflection related to self and others (60–63), less SMFG activation in misophonia patients during successful compared to failed inhibition additionally might represent either (negative) self-reflection following an inhibition error or, conversely, its absence following inhibition success. The positive correlation between this pattern of superior medial frontal gyrus activation and stop signal delays implies that such (absence of) self-reflection may lie at the foundation of the response bias.

On the other hand, Pearson et al. (64) have proposed a general function for the PCC in detection of changes in the environment, in turn informing the DLPFC about potential necessity for adjustment of behavioral policy. Hence, patients' lack of inhibition success-related PCC activation could reflect their failure to detect the impact of inhibition success on speed-accuracy balance. This view is in line with the second interpretation of left DLPFC behavior stated above.

### Strengths and Limitations

Despite the current sample being of regular size for clinical neuroimaging research, modest sample size limits the generalizability of the results and power of the analyses and could have resulted in the behavioral group difference(s) failing to remain significant after multiple comparison correction. Nevertheless, we found significant differences in neuronal activation between patients and controls after appropriate controlling for multiple comparisons.

The present task contained visual stimuli unrelated to patients' clinical features, thus probing response inhibition in the absence of a misophonic reaction. This might have resulted in the current study not having found a potentially clinically relevant endophenotype. It is plausible that response inhibition is solely impaired in context of misophonic triggers, considering that Kumar and colleagues have linked abnormal patterns of neural activity and functional connectivity to the misophonic reaction (5). Various psychiatric disorders show aberrant response inhibition, albeit often with small effect sizes (12), thus contradicting that impaired response inhibition is a primary symptom in itself.

Furthermore, the task lacked variable jitter between trials, something often implemented to avoid anticipation of subsequent go-signals. However, we checked for negative and extremely short RTs, which, in combination with deviation on other behavioral measures, resulted in the exclusion of two controls and two patients from analysis.

It appears we did not probe response inhibition ability in the intended manner, thereby restricting statements about it. However, we might have captured an endophenotype that more genuinely reflects the biological basis of misophonia and that might support recognition of misophonia as a distinct disorder as well as help with its classification within current systems.

### Future Directions

First and foremost, replication of current results is necessary. In addition, future studies could address whether the putative response bias of misophonia patients on the SST could be due to an inability to maintain speed-accuracy balance similarly to controls or if it is a choice (whether explicit or not). For example, this behavior could result from disrupted information accumulation, as already suggested above, or cognitive inflexibility. Another possibility is aberrant error detection, which might lead to implicit and/or explicit biases towards making mistakes. Lastly, research could further address the role of personality in this behavior. More insight into these issues would aid interpretation of the current functional neuroimaging results.

Furthermore, since we found different default mode network activation in patients during the SST, it would be interesting to consider other network characteristics, such as functional connectivity within the default mode network and its coupling with the salience and central executive networks. The latter has previously been found distorted in patients with OCD, schizophrenia, depression, anxiety, dementia, and autism (65, 66).

Yet, considering still so little is known about misophonia and there is need for clinically useful insights, future research might want to focus on other, more symptom-related, aspects of misophonia. Within the context of response inhibition, future research could, for example, adopt an auditory SST which utilizes personalized misophonia trigger sounds in addition to a neutral sound to signal the need for inhibition. Furthermore, considering the variety in trigger sounds—possibly in combination with visual stimulation—and emotions and cognitions constituting the misophonic reaction, it is possible that for misophonia, like OCD, multiple subtypes can be distinguished on the basis of core symptoms. This requires future research with considerably larger sample sizes.

Lastly, we have adopted the in-house developed A-MISO-S—one of the first available misophonia questionnaires—to assess misophonia severity, yet since collection of our data, a variety of misophonia questionnaires has been developed. For future research, it is of great importance that such tools are validated.

### Conclusions

In conclusion, misophonia patients seem to show a marginal response bias on the SST, favoring accuracy over speed. This implies perfectionism and inflexible rule-based rather than ill-considered and prematurely expressed behavior, thus resembling compulsive rather than impulsive behavior. Moreover, brain activations were in line with patients, compared to controls, engaging more cognitive control for slowing responses in service of inhibition success rather than during response inhibition itself, like healthy subjects do, while employing more attentional/computational resources for successful inhibition.

## Ethics Statement

The study was approved by the Medical Ethics Committee of the AMC, Amsterdam, and all participants were explained the nature of the experimental procedures and subsequently provided written informed consent prior to inclusion in the study. This study was carried out in accordance with the recommendations of the Medical Ethics Committee of the AMC, Amsterdam, with written informed consent from all subjects. All subjects gave written informed consent in accordance with the Declaration of Helsinki. The protocol was approved by the Medical Ethics Committee of the AMC, Amsterdam.

## Author Contributions

AS, GW, and DD have been involved in the conception and design of the study. AS has been involved in the acquisition of data. GW and DS have been involved in data analysis and subsequent interpretation of the results. All co-authors have critically reviewed the manuscript. NE has been involved in data acquisition, analysis, and interpretation, as well as drafting of the manuscript.

## Conflict of Interest

The authors declare that the research was conducted in the absence of any commercial or financial relationships that could be construed as a potential conflict of interest.
