# Supplementary material for: Neural Basis of Response Bias on the Stop Signal Task in Misophonia
Source: Front Psychiatry. 2019 Oct 23;10:765. doi: 10.3389/fpsyt.2019.00765 (PMC6819955; doi:10.3389/fpsyt.2019.00765)
Supplement: Supplementary file 1 [file DataSheet_1.pdf]

Supplementary Material

Neural Basis of Response Bias on the Stop Signal Task in Misophonia

Nadine Eijsker*, Arjan Schröder, Dirk J.A. Smit, Guido van Wingen, Damiaan Denys

*** Correspondence: Nadine Eijsker:** nadine.eijsker@gmail.com

Supplementary Table 1. fMRI main effects from whole-brain voxel-wise analysis with a significance criterion of FWE-corrected $p < .05$ and a cluster-defining threshold of $p < 0.001$. Clusters with a p-value between .05 and .10 are printed in italic.

Region	Side	*p*-value	Voxels in cluster	Peak z-value	MNI coordinates x	y	z
Successful inhibition > correct go							
Middle occipital gyrus – inferior temporal gyrus – angular gyrus	R	< .001	8881	> 8	36	-86	0
Middle/inferior occipital gyrus – fusiform gyrus	L	< .001	5728	> 8	-34	-88	0
Insula	L	< .001	881	> 8	-32	18	-8
Insula – inferior frontal gyrus – superior frontal gyrus	R	< .001	5668	7.76	34	22	-4
Middle/superior frontal gyrus	L	.043	270	4.72	-28	-2	50
Correct go > successful inhibition							
Pre-central/post-central gyrus	L	< .001	129494	> 8	-38	-20	64
Caudate nucleus	R	< .001	1039	5.95	20	26	14
Insula – superior temporal gyrus - rolandic operculum	L	< .001	2395	5.88	-46	-10	2
Cuneus	L+R	.002	584	5.38	-8	-98	16
Rolandic operculum – superior temporal gyrus	R	< .001	1531	4.89	44	-20	12
Cerebellum	R	.009	410	4.80	30	-84	-28
Caudate nucleus	L	.004	497	4.67	-18	2	24
Pre-central gyrus	R	.029	304	4.40	38	-20	66
Failed inhibition > correct go							
Insula - inferior frontal gyrus	L	< .001	1740	> 8	-34	18	-10
Insula – pre-central gyrus	R	< .001	3471	> 8	38	22	0
Medial cingulate cortex – posterior medial frontal cortex	L+R	< .001	4210	> 8	8	26	32
Supramarginal gyrus – middle/inferior occipital gyrus	L	< .001	3387	6.57	-60	-42	30
Superior temporal gyrus	R	< .001	4495	6.33	62	-42	22
Pallidum – (bilateral) pons	L	< .001	1471	6.13	-12	4	2
Calcarine gyrus	L	.032	296	4.45	-18	-64	4
Correct go > failed inhibition							
(Right) caudate nucleus - inferior frontal gyrus	L+R	< .001	4245	6.28	22	26	14
Cerebellum	R	.002	537	6.07	38	-76	-36
Angular gyrus	L	.001	677	5.44	-42	-70	30
Superior frontal gyrus - dorsolateral prefrontal cortex	R	.006	441	4.81	20	42	44

Superior temporal gyrus	R	.047	262	4.67	60	-4	-6
Superior occipital gyrus	R	.068	233	4.44	16	-94	20
Posterior cingulate cortex – precuneus	L	< .001	952	4.45	0	-46	34
Successful > failed inhibition							
Middle orbital gyrus – inferior frontal gyrus	L	.018	348	5.74	-34	44	-10
Angular gyrus - post-central gyrus	R	< .001	2754	4.83	28	-56	40
Putamen	R	.037	284	4.81	26	2	-6
Inferior parietal lobule – angular gyrus	L	.067	234	4.31	-46	-58	44
Inferior/middle temporal gyrus	R	.051	256	4.08	46	-66	-4
Calcarine gyrus – middle/inferior occipital gyrus	R	.028	307	4.00	18	-96	-4
Failed > successful inhibition							
Anterior/medial cingulate cortex	L+R	< .001	3845	7.77	-6	22	32
Pre-central gyrus – middle frontal gyrus	R	< .001	754	6.29	46	-12	38
Post-to-pre-central gyrus	L	< .001	1374	6.03	-56	-16	44
Post-central gyrus – temporal pole – superior temporal gyrus	L	< .001	2765	5.79	-60	4	14
Calcarine gyrus	L+R	< .001	1301	4.92	-18	-64	4
Rolandic operculum – insula	R	< .001	922	4.77	58	6	10

Supplementary table 2. fMRI simple effects. Clusters with a p-value between .05 and .10 are printed in italic.

Region		Side	ROI	p-value	Voxels in cluster	Peak z-value	MNI coordinates x	y	z
**Dorsolateral prefrontal cortex**									
Controls	successful inhibition > correct go	L	Control	< .001	-	4.92	-38	30	32
Patients	correct go > successful inhibition	L	Control	*.068*	-	3.14	-24	34	32
**Superior Medial Prefrontal Cortex**									
Patients	failed > successful inhibition	L(+R)		< .001	3723	6.93	-6	24	32
**Posterior cingulate cortex**									
Controls	successful > failed inhibition	(L+)R		.048	2241	4.57	2	-44	32

Supplementary table 3. Brain-behavior correlations: Eigenvariates of BOLD response from clusters showing a group by condition interaction correlated with stop signal delay (SSD).

**Correlated measures**		**Correlation over groups**		**Between-group statistical test**	
		**r**	***p*-value**	**Fisher's Z**	***p*-value**
L DLPFC *correct going minus successful inhibition*	SSD	0.29	.062	0.67	.50
SMFG *failed minus successful inhibition*	SSD	0.38	.012¶	0.23	.82
PCC *failed minus successful inhibition*	SSD	0.29	.066	0.57	.57

¶ Significant after TCH correction with Sankoh et al. (1997) modification (p<.02)
